# Steroid-induced hypokalemic periodic paralysis: a case report and literature review

**DOI:** 10.1186/s12882-023-03131-3

**Published:** 2023-03-24

**Authors:** Haw-Ting Tai, Po-Tsang Lee, Shih-Hsiang Ou

**Affiliations:** 1 Division of Nephrology, Department of Internal Medicine, Kaohsiung Veterans General Hospital, Kaohsiung, Taiwan; 2 Faculty of Medicine, School of Medicine, National Yang Ming Chiao Tung University, Taipei, Taiwan; 3 Division of Nephrology, Department of Internal Medicine, Pingtung Veterans General Hospital, No. 1, Rongzong E. Rd., Pingtung County 900010 Pingtung City, Taiwan (R.O.C.)

**Keywords:** Hypokalemic periodic paralysis, Channelopathy, Glucocorticoids

## Abstract

**Background:**

Hypokalemic periodic paralysis (HPP) is a rare channelopathy characterized by episodic attacks of acute muscle weakness concomitant with hypokalemia. The etiology of hypokalemia is the shift of potassium into the cells, and the clinical symptoms resolve when potassium starts to leak back to the serum. Most of the time, the underlying ion channel defects are well compensated, and an additional trigger is often required to initiate an attack. Well-known trigger factors include carbohydrate-rich meals, exercise followed by rest, stress, cold weather, and alcohol consumption.

**Case presentation:**

Here, we present the case of a 26-year-old Asian man who suffered from an acute onset of bilateral lower limb weakness with hypokalemia following dexamethasone injection. He was diagnosed with HPP.

**Conclusions:**

We would like to remind physicians to think of steroids as an unusual precipitating factor while managing patients with HPP, per results of this case study.

## Background

Hypokalemic periodic paralysis (HPP) is a rare neuromuscular disorder regarded as an ion-channel disease [1, 2]. Attacks lead to the sudden onset of muscle flaccidity associated with low serum potassium levels. Proximal muscles are more affected than distal muscles; ocular, bulbar, and respiratory muscles are usually mildly involved or spared [3]. Muscle tone and deep tendon reflexes decrease during the attack; however, consciousness is preserved. Although the duration usually lasts several hours with spontaneous recovery, it sometimes ranges from minutes to several days. The first attack often begins in adolescence and usually occurs in more than one episode in a lifetime. With increasing age, some patients may develop muscle degeneration and permanent weakness.

Most of the time, the underlying ion-channel defects are well compensated, and an additional trigger is often required for attacks. Some trigger factors of HPP include carbohydrate-rich meals, exercise followed by rest, and stress (excitement or fear), which are associated with increased release of insulin or epinephrine [1]. Cold weather, alcohol, night rest, beta-adrenergic agonists, and medications that can induce hypokalemia might also induce attacks [4].

Glucocorticoids have multiple modes of action mediated via genomic and non-genomic pathways [5]. The genomic actions of glucocorticoids are mediated by its receptor, influencing gene expression in several ways. Most of its anti-inflammatory activity stems from the transrepression of NF-κB and activator protein-1 (AP-1) [6]. Glucocorticoids are widely used in clinical scenarios such as asthma, chronic obstructive pulmonary disease (COPD), rheumatoid arthritis (RA), and other autoimmune diseases. They are ever considered a triggering factor of HPP attacks; however, limited cases of these agents precipitating paralysis have been reported [7–11]. The role and mechanism of glucocorticoids in inducing HPP attacks are crucial as they are widely used.

This article reports the case of a 26-year-old Asian man who experienced muscle weakness due to HPP following dexamethasone administration. Therefore, we describe the rare association between steroid use and acute limb weakness.

## Case report

A 26-year-old Asian man was transported to the emergency department via ambulance due to bilateral lower limb weakness. He had no relevant medical history except for a diagnosis of aseptic meningitis more than 10 years ago without sequelae. The patient was a construction worker who performed heavy labor. He stated that he had suffered from intermittent muscle cramping over the right shoulder radiating to back for a week, and he visited a local traditional medicine practitioner a day before the emergency department visit. Non-steroidal anti-inflammatory drugs and another unknown agent were injected. The muscle cramping improved before he slept. However, acute limb weakness occurred after the patient woke up in the morning. He had difficulty standing and fell, resulting in upper limb contusions.

Upon arrival at our emergency department, the tympanic membrane temperature was 38.1°C, with normotension and no other abnormalities in vital signs. In addition, a review of the system showed no loss of consciousness, limb or trunk pain, upper respiratory symptoms, vomiting, diarrhea, or heavy sweating. He had no regular consumption of alcohol or smoking habits, and his family history included only hypertension and diabetes mellitus of his grandparents, without diseases related to hypokalemia or muscle weakness.

The patient was oriented with a good mental health status and appeared to be well nourished. Physical examination showed decreased muscle power of the bilateral lower limbs, mainly proximal, and both scored 2/5, which progressed to 1/5. The deep tendon reflex of the bilateral knees and sensation of the lower limbs were intact, and the muscle power of the upper limbs was not affected. There was also no Babinski sign elicited on either side. Blood tests, including complete cell count, blood sugar, and a biochemical panel, were normal, except for white blood cells (10,800/μL), potassium (1.7 mEq/L), and creatine phosphokinase (178 U/L). Urinalysis was normal. However, electrocardiogram showed a flattened T wave and exhibited U waves in the precordial leads V1–V3.

Oral and intravenous potassium supplements with 40 mEq was administered, and the serum potassium level result was still 2.0 mEq/L the following morning. We then administered another 60 mEq of potassium, and upon assessment, the muscle power of the patient’s bilateral lower limbs improved in the afternoon (4/5). He was admitted to the ward and was administered an additional 20 mEq of intravenous potassium (a total of 120 mEq since the emergency department); hypokalemia resolved (serum potassium 4.0 mEq/L), and the patient could walk steadily. Because his paralysis improved rapidly after hypokalemia correction and myopathies were less likely, electromyography was not done during admission.

The urinary potassium excretion of the patient was low (urine potassium/creatinine ratio is 1.5 mmol/mmol) and there was no evidence of potassium loss from gastrointestinal tract. He also denied potassium-shifting or potassium-wasting medication use, such as insulin, beta-agonists, thyroxine, or diuretics. The levels of serum aldosterone (109.5 pg/mL), renin (34.98 pg/mL), and free T_4_ (1.14 ng/dL) were borderline normal; however, elevated levels of serum thyroid stimulating hormone (TSH) (5.91 uIU/mL) were noted. Arterial blood gas analysis was normal. Hypokalemic periodic paralysis was considered as the clinical impression because of rapid normalization of potassium levels and improvement of weakness. However, the patient did not receive further genetic tests due to personal reasons.

Furthermore, the patient denied having much carbohydrate-rich food intake or strenuous exercise before the attack. His family contacted the local clinic which disclosed that it had administered 5 mg dexamethasone through intramuscular injection. His serum cortisol level was <1.00 μg/dL and 4.39 μg/dL at 2 and 4 days after the injection, respectively. The final diagnosis was HPP of the non-familial type, suspected to be induced by dexamethasone injection. Finally, he was discharged without further sequela.

One year later, he came back to our emergency department due to hypokalemia-induced paralysis after eating a big carbohydrate-rich meal. We advised him to avoid using corticosteroids and eating carbohydrate-rich food.

## Discussion and conclusion

We present the case of a 26-year-old Asian man with acute onset of bilateral symmetrical lower limb weakness with concomitant hypokalemia. HPP was diagnosed due to the symptoms, short duration, and rapid improvement with potassium supplementation. The triggering factor of HPP was likely iatrogenic dexamethasone injection.

HPP is considered to be a channelopathy due to defective ion channels, mainly resulting from mutations in genes encoding the subunit of L-type voltage-dependent calcium channel Cav1.1 (CACNA1S) and skeletal muscle sodium voltage-gated channel Nav1.4 (SCN4A) [12, 13]. The disease is usually autosomal dominant but sometimes occurs sporadically. There are also cases with none of these mutations identified, and a few with other possibly related mutations, such as R1128H/C mutation in the NaV1.4 sodium channel [14]. Men are likely to experience symptoms more often than women because of the difference in penetrance [15].

Triggers are important for inducing a paralysis attack. After initial exposure to triggers, the activity and number of Na-K-ATPases on the cell membrane changes, causing mild potassium influx into cells. Paradoxical depolarization of the skeletal membrane potential occurs, exaggerating extracellular hypokalemia with a smaller efflux of potassium, inactive sodium channels, loss of excitability, and muscle weakness [12, 16]. Therefore, the identification of specific triggers and their prevention are important and advised for patients with HPP. Beta-adrenergic agonists, insulin, or other medications that induce hypokalemia are possible triggers. Carbohydrate-rich meals, exercise followed by rest, and stress (or excitement/fear/cold) exposure might directly increase sympathetic tone by releasing more catecholamines, indirectly cause hyperinsulinemia, and then stimulate the activity of skeletal muscle Na-K-ATPase, resulting in hypokalemia and HPP attack.

Glucocorticoids are rarely considered as possible triggers of HPP attacks, and the mechanism is not well understood now. Steroids have been reported to cause direct myopathy, and their inherent mineralocorticoid effect can induce renal loss of potassium [17]. However, this adverse effect usually occurs with long-term use. Several possible mechanisms have been proposed for explanation of steroid induced HPP attack. First, glucocorticoids can cause insulin resistance, which results in hyperglycemia and hyperinsulinemia [18]. Insulin increases the activity and number of Na-K-ATPases, and thereby, the intracellular shift of serum potassium [19]. Insulin also inhibits the current of the inward rectifier potassium channel (Kir) and leads to a more depolarized membrane potential [20]. Second, glucocorticoids can upregulate beta-2 receptors on the cell membrane, and the interaction between beta-2 receptors and catecholamines also has a stimulatory effect on the Na-K-ATPase [21]. Third, glucocorticoids directly regulate transcription of the Na-K-ATPase, increasing the excitement potential of skeletal cell membranes [22]. However, the severity of hypokalemia after steroid use varies between individuals. Further, the incidence of HPP attack after steroid exposure is not common among patients. In our opinion, since steroid injection rarely causes HPP in clinical practices, specific genetic background or environmental factors are required to trigger HPP. Nevertheless, additional basic experiments may be needed to confirm the hypothesis.

Reported cases of HPP after administration of glucocorticoids in the literature and have summarized the cases in Table 1. Of the 17 cases (including our case), 15/17 (88%) were men. The average age at attack after glucocorticoid administration was 24.6 years (except for the missing data of case #6), and four patients had previous episodes of attack in the younger age group. In our observation, most of the reaction times were within 24 hours after exposure. There were diverse in different kinds of glucocorticoid used (prednisolone, methylprednisolone, and dexamethasone), different routes of administration (oral, intravenous, and intramuscular administration), and different doses. However, most cases were based on clinical suspicion and exclusion, lacking definite evidence of a link. If a rechallenge test can be done, it will help to confirm the causal relationship.

**Table 1:** Information on patients who contracted hypokalemic periodic paralysis from systemic glucocorticoid administration

Source	Case	Age at attack (years)	Sex	Lowest serum potassium measured (mmol/L)	Steroid, dose and administration	Administration-to-attack time	Other episodes	Family history	Gene mutation
Our case	1	26	M	1.7	Dexamethasone, IM	Within 24 hours	First	No	N/A
Arzel-Hézode M. et al. (2009) [10]	2	36	M	2.7	Methylprednisolone, 48 mg PO	Within 24 hours	First	Yes	SCN4A, R675Q
	3	23	M	1.4	Methylprednisolone, 48 mg PO	Within 24 hours	One previous	No	SCN4A, R675W
	4	26	M	1.9	Methylprednisolone, 120 mg IV	Within 24 hours	First	No	No mutation found
	5	25	M	2.49	Methylprednisolone, 120 mg IV	Within 24 hours	First	N/A	No mutation found
	6	N/A	M	2.1	Prednisone, 60 mg PO	Within 24 hours	First	No	No mutation found
	7	20	M	2	Prednisone, 60 mg PO	N/A	First	Yes	No mutation found
	8	31	M	2	Methylprednisolone, 100 mg IV	Within 24 hours	One previous	No	No mutation found
	9	16	M	2.7	Prednisolone, 60 mg PO	Within 24 hours	First	Yes	SCN4A, R672H
	10	15	F	N/A	Methylprednisolone, 16 mg PO	N/A	First	No	CACNA1, R528H
	11	16	M	2.2	Prednisolone, 80 mg PO	Within 24 hours	First	No	Kir, KCNJ2, C54F
	12	27	M	2.4	Prednisone, 60 mg PO	Within 24 hours	First	No	SCN4A, R1451C
	13	35	M	0.8	Prednisolone, 80 mg PO	8 days	One previous	Yes	CACNA1, R528H
Corey M. Teagarden et al. (2011) [7]	14	23	F	1.6	Betamethasone, 12 mg IM for two times	Within 24 hours	First episode	No	N/A
J. Casey Elkins (2019) [8]	15	27	M	1.5	Dexamethasone, IV	N/A	Two previous	N/A	N/A
S.R. Harter et al. (2019) [9]	16	33	M	1.9	Methandienone, a C17-alpha alkylated steroid Decadron, 250 mg IM daily	N/A	First	N/A	N/A
Ana Catarina Carvalho et al. (2019) [23]	17	15	M	1.9	Methylprednisolone	N/A	First	Yes	N/A

*M* Male, *F* Female, *IM* Intramuscular, *IV* Intravenous, *PO* Per os

We report an unusual case of HPP with an attack following the administration of glucocorticoids. The importance of our finding is to remind physicians that HPP should be included in the differential diagnosis if sudden onset of generalized flaccid muscle occurs after glucocorticoid administration. Moreover, glucocorticoids should be carefully used in patients with HPP because medication may be one of the possible triggers.

## Data Availability

The data used to support the findings of this study are available from the corresponding author upon reasonable request.

## Abbreviations

HPP: Hypokalemic periodic paralysis
AP-1: Activator protein-1
COPD: Chronic obstructive pulmonary disease
RA: Rheumatoid arthritis
TSH: Thyroid stimulating hormone
Kir: Inward rectifier potassium channel
